# Two h-Index Benchmarks for Evaluating the Publication Performance of Medical Informatics Researchers

**DOI:** 10.2196/jmir.2177

**Published:** 2012-10-18

**Authors:** Khaled El Emam, Luk Arbuckle, Elizabeth Jonker, Kevin Anderson

**Affiliations:** ^1^Electronic Health Information LaboratoryChildren's Hospital of Eastern Ontario Research InstituteOttawa, ONCanada; ^2^Department of PediatricsFaculty of MedicineUniversity of OttawaOttawa, ONCanada

**Keywords:** h-Index, medical informatics, bibliometrics, evaluation, research output

## Abstract

**Background:**

The *h*-index is a commonly used metric for evaluating the publication performance of researchers. However, in a multidisciplinary field such as medical informatics, interpreting the *h*-index is a challenge because researchers tend to have diverse home disciplines, ranging from clinical areas to computer science, basic science, and the social sciences, each with different publication performance profiles.

**Objective:**

To construct a reference standard for interpreting the *h*-index of medical informatics researchers based on the performance of their peers.

**Methods:**

Using a sample of authors with articles published over the 5-year period 2006–2011 in the 2 top journals in medical informatics (as determined by impact factor), we computed their *h*-index using the Scopus database. Percentiles were computed to create a 6-level benchmark, similar in scheme to one used by the US National Science Foundation, and a 10-level benchmark.

**Results:**

The 2 benchmarks can be used to place medical informatics researchers in an ordered category based on the performance of their peers. A validation exercise mapped the benchmark levels to the ranks of medical informatics academic faculty in the United States. The 10-level benchmark tracked academic rank better (with no ties) and is therefore more suitable for practical use.

**Conclusions:**

Our 10-level benchmark provides an objective basis to evaluate and compare the publication performance of medical informatics researchers with that of their peers using the *h*-index.

## Introduction

Publication metrics, such as the impact factor of journals and the number of citations to papers, are often used directly or indirectly to evaluate the performance of researchers for hiring, promotion, and funding decisions [1-7]. For example, the US National Institutes of Health has developed an electronic Scientific Portfolio Assistant linked to publication metrics [8,9] (such as impact factor and number of citations) and is used by National Institutes of Health staff to “make close-call funding decisions on individual grants” [10]. Similarly, some Wellcome Trust panels have used impact factor and applicant citation data to make grant funding decisions [7]. Publication metrics are also used to evaluate research institutions [11-13] and assess the impact of biomedical research funding policies and programs [9,10,14-16].

Direct comparisons of researchers from different backgrounds and disciplines on publication metrics can be quite misleading [17-21]. This can be a challenge for medical informatics in that it is generally considered a multidisciplinary field [22-26]. For example, one analysis of the US National Library of Medicine’s Medical Subject Headings (MeSH) that were used for medical informatics articles identified clusters indexed by terms related to the science and art of medicine, molecular genetics, statistical analysis, immunology, and biochemical communications [25]. A citation analysis of medical informatics articles found that work in general medicine journals was often cited [22].

The comparability problem is demonstrated in Table 1, which shows the average number of citations per paper over a 10-year period for a variety of disciplines [18]. There is an almost 10-fold difference in the average number of citations per paper between a researcher in molecular biology and genetics, and a researcher in computer science. Consider a computer scientist who, with a mean of 5 citations to her papers, would be considered an above-average performer on that metric (for a computer scientist) but, when compared with a basic scientist with average performance she would be assessed quite poorly. Given that both a computer and a basic scientist can be medical informatics researchers and possibly affiliated with the same institution or department, there is a need for methods to evaluate and interpret their publication metrics that allow fair and meaningful comparisons with their medical informatics peers.

**Table 1:** Average number of citations per paper between 1995 and 2005 by discipline [18].

Discipline	Average citations per paper
Clinical medicine	10.58
Computer science	2.49
Economics and business	4.17
Engineering	3.17
Mathematics	2.66
Molecular biology and genetics	24.57
Neuroscience and behavior	16.41
Pharmacology and toxicology	9.4
Psychiatry and psychology	8.24
Social sciences, general	3.46

### The h-Index

One of the more-commonly used metrics to evaluate the publication performance of researchers is the *h*-index [27]. This was first proposed and defined by Hirsch in 2005 as follows: “A scientist has an index *h *if *h *of his or her *N* *p *papers have at least *h *citations each and the other (*N* *p *– *h*) papers have ≤*h *citations each” [27]. Hirsh designed the *h*-index to avoid the problems of other common bibliometrics, such as the total number of papers, total number of citations, number of citations per paper, number of significant papers with >*y *citations (*y *is determined by the evaluator), and number of citations to each of the *q *most-cited papers (*q *is determined by the evaluator). The *h*-index measures the impact of an individual’s output rather than the volume, controls for the effect of a small number of highly cited papers, rewards consistent output, and is less arbitrary than measures for number of significant papers or number of citations to the *q *most-cited papers [27]. Its ease of use is also a benefit, as it is a single number that is simple to calculate using readily available databases that provide citation counts. Another advantage is that the *h*-index has been shown to predict the impact a researcher will make in the future. In a 2007 study by Hirsh, the predictive power of *h *was compared with that of 3 other bibliometrics: total number of papers, total number of citations, and mean number of citations per paper [28]. It was found that authors’ *h*-index scores after the first 12 years of publication were best able to predict performance in the subsequent 12-year period, as well as cumulative achievement over the entire 24-year period. A high correlation has also been found between an individual’s *h*-index and his or her receipt of academic awards, appointments, and funding [17,29]. A person’s *h*-index has also been found to be relatively unaffected by normal citation record errors—a 2010 review reported that *h*-scores before and after the correction of errors remained stable [29].

Many variations of and alternatives to the *h*-index have been proposed since 2005 [17,29-31], for example, to give more weight to highly cited papers [30], incorporating the variables of total number and age of citations [29] and allowing comparability across disciplines [31].

However, many of the subsequent variations proposed have been shown to be highly correlated with the *h*-index, and hence do not provide much additional information [32], and each variation increases the complexity and computational difficulty of the metric. As noted in a recent review, “many *h*-index variations, although being designed to overcome some of its supposed limitations, do indeed correlate quite heavily. This fact has made some researchers think that there is probably no need to introduce more *h*-index variations if it is not possible to prove that they are not redundant in real examples” [17]. Aided by the inclusion of automated *h*-index calculators in popular interdisciplinary databases, use of the *h*-index continues to grow [17].

A more-detailed critical review of the *h*-index and its measurement is provided in Multimedia Appendix 1.

### Uses and Interpretation of the h-Index

In the basic, natural, and applied sciences, there has been a trend toward objective performance evaluations of researchers for hiring, promotion, and funding decisions using bibliometrics, including the *h*-index [1-6]. In the health sciences, individuals, departments, and institutions have been compared using their *h*-index scores [21,33-39]. The *h*-index has also been used in medical informatics to evaluate the quality of panel sessions at the annual American Medical Informatics Association symposium [40] and to evaluate the national influence of medical informatics research [41].

Traditionally, subjective peer evaluations have been used as the main method to evaluate researcher performance. There is evidence that the *h*-index scores correlate well with peer assessments [42-45]. However, a case can be made for using the *h*-index to inform the peer-review decision-making process, which can arguably enhance interreviewer reliability (see [46]).

Proper interpretation of publication metrics requires a robust reference standard [47], and there is none for the *h*-index in the area of medical informatics. Given the relative advantages of the *h*-index as an objective measure of scientific output, such a standard is needed for the *h*-index to be used effectively. A defensible standard to interpret the *h*-index can help accelerate its adoption in medical informatics and allow objective, repeatable, and fair evaluations and comparisons of researchers.

In the past, different types of reference standards for the *h*-index have been constructed in other disciplines. Examples include using the mean for multiple scientific disciplines [18], by rank of radiology academics [33], by rank of neurosurgery academics [21], by comparison with chairs of medical departments in US medical schools [34], as a median for academic anesthesiologists [38,39], by rank of academic anesthesiologists [37], and by rank for academic urologists [48].

Because citation and publication distributions are known to be heavily skewed [49,50], reference standards based on percentiles have been recommended [51]. In this study we developed 2 percentile-based benchmarks for interpreting the value of the *h*-index for researchers who publish in medical informatics journals and validated the benchmarks using an independent measure of performance.

## Methods

Our objective was to develop appropriate *h*-index benchmarks for researchers who publish in medical informatics journals.

### Requirements for Benchmarks

We considered the following as requirements to maximize the utility of the benchmarks. We therefore used them to guide the methodological decisions made during their construction:

R1. The benchmarks should allow for the evaluation of researchers’ performance at multiple stages in their careers. This means that the benchmarks should have sufficient granularity and variation to reflect the performance of early career investigators as well as more-established researchers.

R2. The benchmarks need to be contemporary, reflecting the performance of researchers at this point of time rather than serving as a historical analytical tool.

R3. The benchmarks should reflect the performance of researchers who publish influential work rather than work that does not get cited often.

We describe below how we made the tradeoffs necessary to meet these requirements.

### Author Sampling Frame

We intended the benchmarks to apply to individuals who are considered *medical informatics researchers*. One approach to identifying medical informatics researchers is to use a subset of prominent individuals in the field, such as American College of Medical Informatics (ACMI) fellows. This approach has an important disadvantage in that ACMI fellows are not necessarily representative of the rest of the medical informatics researchers and would therefore not meet requirement R1 above because (1) they have higher *h*-index values than other researchers, and (2) they constitute a very small fraction of all medical informatics researchers.

While constructing benchmarks based only on ACMI fellows would meet requirement R3, this group has higher *h*-index values and would not be a good representation of all medical informatics researchers. To confirm this, we compared the *h*-index values for fellows with those who were not fellows in a simple random sample of 430 authors from all authors in the top (by impact factor) 2 medical informatics journals as classified by *Journal Citation Reports*: the *Journal of Medical Internet Research *(JMIR) and the *Journal of the American Medical Informatics Association *(JAMIA), with 5-year impact factors of 4.98 and 3.94, respectively (according to the Web of Knowledge). Fellows had a mean *h*-index value of 16.5 versus 8.8 for the nonfellows. This difference was statistically significant (*P *< .05 for a 2-tailed *t *test, and *P *< .05 for a Mann-Whitney nonparametric test).

A very small proportion of authors in medical informatics journals are ACMI fellows. This is because there are only 338 fellows, they do not all publish in medical informatics journals, and many of the fellows are now retired or deceased and are no longer actively publishing. Table 2 shows the percentage of authors in some of the *Journal Citation Reports *medical informatics journals who were ACMI fellows during the period 2006-2011. The first number in the table is the maximum possible and assumes that all ACMI fellows publish in that journal. The second number is based on an exact match of the names of fellows and authors in the journal, and the third number is based on an approximate match of the names using the Jaro-Winkler string comparison distance [52] (with a cut-off of 0.9). The exact match rate is likely to have a higher false-negative rate and the latter, with the approximate matching, a higher false-positive rate. Therefore, the correct match rate is expected to be within these two values. Across all journals, the fellows account for 0.5%-0.68% (using exact and approximate matching, respectively) of all authors.

**Table 2:** Percentage of American College of Medical Informatics fellows who published in some of the *Journal Citation Reports *medical informatics journals over the period 2006–2011.

Journal name	Maximum match (%)	Exact match (%)	Approximate match (%)
*Journal of the American Medical Informatics Association *(JAMIA)	12.8	4.63	5.62
*Journal of Medical Internet Research *(JMIR)	23.5	0.9	1.32
*IEEE Engineering in Medicine and Biology*	37.3	0	0.22
*Artificial Intelligence in Medicine*	28.8	0.94	1.45
*BMC Medical Informatics and Decision Making*	21.5	1.4	2.1
*Computers, Informatics, Nursing*	34.5	1.43	2.14
*Computer Methods and Programs in Biomedicine*	11.5	0.24	0.37
*IEEE Transactions on Information Technology in Biomedicine*	12.7	0.07	0.22
*International Journal of Medical Informatics *(IJMI)	15.5	2.65	3.52
*International Journal of Technology Assessment in Health Care*	21.5	0.06	0.25
*Journal of Biomedical Informatics *(JBI)	16.9	3.4	4.35
*Journal of Medical Systems*	14.8	0.13	0.17
*Medical & Biological Engineering & Computing*	11.3	0	0.1
*Medical Decision Making*	21.4	0.63	1
*Methods of Information in Medicine*	18.7	0.33	2.77
*Statistics in Medicine*	8.1	0.17	0.38
*Statistical Methods in Medical Research*	45.3	0.13	0.4

Another approach to constructing a sampling frame is to identify all authors who publish in medical informatics journals over a specified time period and consider them to be medical informatics researchers. Various approaches have been used in the literature to identify a core set of medical informatics journals, which we review below.

A bottom-up method uses index terms in article databases to identify journals [22,23,53,54]. For example, some studies used MeSH terms for medical informatics concepts. However, a recent analysis found that the journals that published the majority of papers classified in this way had central concepts outside of medical informatics and were “not typically identified as medical informatics-specific journals,” such as physics, imaging, and engineering journals [23]. Therefore, this approach would not have strong face validity and is unlikely to be intuitively convincing.

A variant of that approach is to identify the journals with the relatively most-cited articles that are indexed or classified under medical informatics [22]. However, many of the journals with the most-cited articles were general medicine or general science journals, since these journals tend to have quite high average citations per article. A survey of ACMI fellows found that general medicine and general science journals ranked lower in terms of readership than did journals more typically associated with medical informatics [55]. Again, our benchmark would not pass the face validity test if it were based on publications that have a low readership among the most experienced members of the community.

Some authors subjectively decide on an initial set of medical informatics journals to study [24-26,55,56] or ask ACMI fellows to rank or rate journals [55,57]. Others use existing classifications of journals, such as the *Journal Citation Reports *[58]. Sometimes multiple approaches are used [57]. The journals in which prominent members of the community publish have also been used as the core set of medical informatics journals, such as the most-cited ACMI fellows [58].

Our approach to identifying source journals for selecting medical informatics researchers was informed by the methods used in the literature. We selected the list of journals in the *Journal Citation Reports *in the medical informatics category. This is consistent with previous approaches [57,58]. We identified the top-2 ranked journals by impact factor at the time of writing: JMIR and JAMIA. If we had considered other definitions of “core medical informatics journals” [24,57], these 2 journals would still have had the highest impact factors among journals in those sets.

We considered all authors who published more than 1 article over the 2006–2011 period in any of the 17 journals in Table 2. Approximately 70%–77% (using exact and approximate matching, respectively) of these JMIR and JAMIA authors had also published at least one paper in one of the other 15 journals. While the choice of JMIR and JAMIA authors seemingly limited our analysis to those who published the articles that were most cited, in fact there is still significant community overlap with other journals.

By defining the sampling frame to consist of all authors in JMIR and JAMIA, plus the overlap in authorship with other journals, we met requirement R1. Requirement R3 was also met because these 2 journals have the highest impact factors.

### Sampling Period

We considered the *h-*index of authors who had published in the 5-year period 2006-2011 in these 2 journals: JMIR 2006;8(1) to 2011;13(2) and JAMIA 2006;13(1) to 2011;18(5). We chose the 5-year period because it is quite common for studies evaluating scholars, institutions, and programs to examine the previous 5 years’ data on publications [59-68], and previous studies assessing the structure of the medical informatics literature and community have often used 5-year intervals [26,58]. In addition, a longer period would likely include more researchers who may no longer be as active in the field, hence reducing the benchmarks’ representativeness of current researchers, and would therefore not meet requirement R2 above.

### Author Order

In addition to constructing a benchmark based on all authors within the sampling frame and sampling period, we could construct benchmarks based on first authors only. However, there is a lack of consistency across disciplines in how authors are ordered. For example, in some cases authors are ordered alphabetically or by the extent of contribution. This makes it difficult to determine when a first-author benchmark should be used. Furthermore, there is evidence of a strong correlation between the *h*-index values based on being a first author and those ignoring author order [69]. We therefore constructed benchmarks ignoring author order.

### Benchmark Levels

In general, a reference standard for publication metrics using percentiles has been recommended [70], and specifically one based on deciles [71]. We refer to a decile benchmark as PR10. A PR10 benchmark provides a 10-level scale based on deciles, where a level 10 means that a researcher is in the top 10% among his or her peers in terms of his or her *h*-index value, and a level 1 means that a researcher is in the bottom 10% among his or her peers. We deemed benchmarks with fewer levels, such as 5 levels based on quintiles, to be too coarse, as they would provide inadequate granularity to assess researchers’ publication output as they move through their career stages and would therefore not meet requirement R1.

Another evaluation scheme used in the Science and Engineering Indicators by the US National Science Foundation has only 6 percentile levels, which we refer to as PR6 [72]. PR6 focuses on authors with *h*-index values that are higher than the median and on benchmarking more-established researchers.

### Calculation of h-Index

For our computation of the *h*-index we used Scopus. We manually checked the identity of all authors in Scopus by confirming their affiliation and comparing their listed publications with personal or academic websites. Scopus also uses specific algorithms [73] to combine all references from each author under a unique ID, which means that the time needed to manually match references to author names is reduced.

Furthermore, Scopus has been shown to have a more-accurate automated calculator for the *h-*index (vs Web of Science]) [74], to include more peer-reviewed conference proceedings than Web of Science [75] and to avoid the problems of duplicate records and false-positives associated with Google Scholar [74-76].

The *h*-index not only is computed from articles published in the 2 top medical informatics journals (JMIR and JAMIA) from which we sampled authors, but also covers all publications by these researchers in all journals and conferences that are indexed, going back to when indexing started. In the case of Scopus, indexing started from 1996. For example, if selected medical informatics researchers also published papers in general medicine journals in 2000, their *h*-index would include their publications in the general medicine journals from that year.

### Sample Size Calculation

We manually computed the *h*-index for medical informatics researchers using Scopus; therefore, rather than computing it for all authors, we decided to estimate it from a random sample. To ensure we had a representative sample of the underlying population, we decided to use a strict quantitative definition of representativeness. This definition also provides insight into the confidence intervals we can expect, based on the uncertainty we chose in sampling, therefore estimating the true unknown distribution of the *h*-index. We will use the PR10 benchmark to describe the method.

Following Sobel and Huyett [77], we selected a random sample of authors based on a nonparametric definition of representativeness. Namely, our sample of authors should be simultaneously representative of the true unknown cumulative distribution *F *of the *h*-index for deciles. That is, we divide the *h*-index into 10 pairwise disjoint subsets that we denote *C*_1_,...,*C*_10_. These subsets are unknown but have probability under *F *greater than zero, and in the case of equiprobable deciles are given by *F*(*C* *_i_*) = 0.10, or *i *= 1,...,10 (where *F *as used here is a probability measure).

For an observed cumulative sample distribution *F* ^*^ *_n_* based on *n *observations, we say that a sample is representative relative to the fixed disjoint subsets *C*_1_,...,*C*_10 _to within the common allowance * ß* ^* ^if we have |*F* ^*^ *_n_*(*C* *_i_*) *– F*(*C* *_i_*)|≤* ß* ^* ^simultaneously for *i *= 1,...,10. The degree of representativeness of *F *under this setup is subsequently defined as *d* ^*^ *_g_*= 1 – 10*ß* ^* ^for deciles. In particular, we consider a common allowance *ß* ^* ^= 0.05/2 = 0.025, as it is a standard threshold used in 1-sided definitions, resulting in a degree of representativeness of *d* ^*^ *_g_*= 0.75 for deciles.

Next we choose a probability *P* ^* ^that the sample will at least have the degree of representativeness of *F *that we selected. That is, *P*(|*F* ^*^ *_n_*(*C* *_i_*) – *F*(*C* *_i_*)| ≤ *ß* ^* ^for all *i *= 1,...,10) ≥ *P* ^*^, where we seek the sample size *n *needed to satisfy this equation. Note that larger sample sizes may not satisfy the inequality, and it is therefore necessary to treat this as an approximation only. For the sample sizes we consider, however, the duplicates in the original author list should decrease the author count such that the sample is inflated enough to meet the probability threshold we desire.

Determining the sample size *n *involves using a multinomial distribution for an infinite population and a hypergeometric distribution for a finite population. Sobel and Huyett [77] provide tables that give the sample size required under a variety of circumstances. Note that for a fixed number of pairwise disjoint subsets *C* *_i_* and fixed allowance *ß* ^*^, the greater the probability *P* ^*^, the greater the required sample size. Let *N *be the population size, *n*_∞ _the sample size for an infinite population, and *n* *_N_* the sample size for a finite population. Then, given *n*_∞ _from the tables, the required sample size can be adjusted using the approximation *n*_∞ _≈ *n* *_N_*((*N *– 1) / (*N *– *n* *_N_*)).

We identified N = 3220 total authors (regardless of position in the author list) and chose a probability of simultaneous representativeness *P* ^* ^= .75 for deciles (*ß* ^* ^= 0.025, *d* ^*^ *_g_*= 0.75), resulting in a minimum sample size of *n*_3220 _= 430 (where *n*_∞ _= 500 for an infinite population).

### Sample of Authors

We extracted article names, author names, and journal edition for JMIR and JAMIA over the 5 years of journal issues. We drew a simple random sample of 430 from the 3220 authors regardless of position in the author list, excluding correspondences or letters, editorials, errata, highlights, and articles without a designated author.

## Results

**Figure 1:**
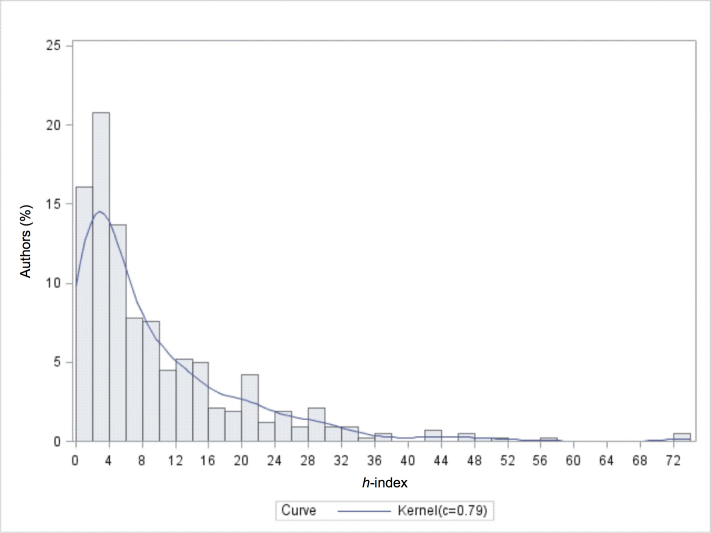
Distribution of *h*-index values from a sample of 430 authors.

The distribution of *h*-index values from our sample is shown in Figure 1. We fitted a kernel density estimate with a normal kernel function and a bandwidth that minimizes the approximate mean integrated square error between the density estimator and the true density. As can be seen, the distributions have a long tail. This means that the maximum *h*-index value will be significantly larger than the 90th percentile value (level 10 in PR10) and the 99th percentile in PR6. For example, the highest value for an author in our sample was 74, but the point estimate of the 90th percentile for our benchmarks was approximately 23. Therefore, authors with values significantly larger than the 90th percentile were all in the top 10% of medical informatics researchers. Since percentiles are a ranking, the actual *h*-index value for a level-10 author can be much higher than the 90th percentile value.

Percentile estimates of the *h*-index of authors who published in JAMIA or JMIR over the 5 years we examined are given in Table 3. Confidence intervals were calculated using the Woodruff method, which inverts the confidence intervals for the estimated distribution function (first proposed Woodruff [78] and further justified by Francisco and Fuller [79], Dorfman and Valliant [80], Sitter and Wu [81] and Chatterjee [82]).

We will consider an example to illustrate how to interpret Table 3. A medical informatics researcher with an *h*-index of 21 could be said to be in the 90th percentile of his or her peers in medical informatics, since that value falls right within the confidence interval. Any *h*-index value as high as 25 would still be in the 90th percentile. A researcher with a value in the range 17–20 is above the 80th percentile (since 17 is larger than the upper confidence limit for the 80th percentile), but not at the 90th percentile. To move beyond the 90th percentile, that researcher would need an *h*-index value of 26 or higher.

Table 4 provides the *h*-index benchmark values that would indicate statistically significant values for each of the levels. These can be used to directly determine the level of researchers based on their *h*-index values.

**Table 3:** *h*-Index percentile estimates for authors published in the *Journal of the American Medical Informatics Association *(JAMIA) or *Journal of Medical Internet Research *(JMIR) over the 5-year period 2006–2011.

PR6^a^	PR10^b^				
Percentile	Estimate	95% CI^c^	Percentile	Estimate	95% CI
<50%			<10%		
50%	4.9	4.2–5.6	10%	0.6	0.5–0.6
75%	12.6	10.9–14.3	20%	1.4	1.1–1.6
90%	22.9	20.2–25.6	30%	2.3	1.9–2.7
95%	28.9	26–31.7	40%	3.4	2.9–3.9
99%	48.5	32.2–64.9	50%	4.9	4.2–5.6
			60%	7.5	6.3–8.7
			70%	10.8	9.3–12.3
			80%	14.8	13.1–16.5
			90%	22.9	20.2–25.6

^a ^6 Percentile-level benchmark.

^b ^Decile benchmark.

^c ^Confidence interval.

**Table 4:** *h*-Index benchmarks for authors published in the *Journal of the American Medical Informatics Association *(JAMIA) or *Journal of Medical Internet Research *(JMIR) over the 5-year period 2006–2011.

Level	Benchmark	
	PR6^a^	PR10^b^
1	0–5	0
2	6–14	1
3	15–25	2
4	26–31	3
5	32–64	4–5
6	≥65	6–8
7		9–12
8		13–16
9		17–25
10		≥26

^a ^6 Percentile-level benchmark.

^b ^Decile benchmark.

To explain how Table 4 was constructed, we take as an example the 50th percentile. Here the upper confidence limit is 5.6. Keeping in mind that *h*-index values can only be integers, any *h*-index value that is 5 or less will be in the lower 50% of all authors. Similarly, if we take the 75th percentile in PR6, any *h*-index value that is 14 or less will be in the bottom 75% of all authors. Consequently, any value that is higher than 5 and equal to or less than 14 will be in the percentile range greater than 50% and less than or equal to 75% (in the third quartile). This is the 6–14 range shown in Table 4.

## Discussion

### Summary

The objective of this study was to construct empirical benchmarks for interpreting the *h*-index for medical informatics researchers. We had specified three requirements to ensure the utility of the benchmarks to a large segment of the community: (1) they allow the tracking of career progress for researchers, (2) the benchmarks should be contemporary, reflecting current work in the field, and (3) the benchmarks should be based on researchers doing work that is cited often. The values we computed represent all publications by authors going back to 1996.

The benchmarks allow for the evaluation of researchers relative to their peers. These peers come from a mix of other disciplines, but they do represent the contemporary medical informatics researcher community.

More precisely, we have provided 2 empirical benchmarks that are slightly different. The first, PR6, uses a National Science Foundation scheme that has 6 percentile levels, and the second, PR10, is a broader 10-level benchmark based on deciles. The PR10 benchmark allows for the evaluation of performance of researchers at early and late stages of their careers. The PR6 benchmark is focused on the top half of performers.

### Validation

To validate the benchmarks, we examined the relationship between the *h*-index and some independent measure or ranking of researcher performance. One option was to rank scientists based on the number of recognitions and awards they have received (eg, the Nobel Prize) and the number of degrees they have received, and then determine whether our benchmarks reflect that ordering [83]. A proxy for achievements of researchers is their academic faculty rank.

We examined whether our benchmarks track the mean *h*-index value of medical informatics faculty in the United States. Several studies have used the mean (or median) *h*-index of faculty ranks to characterize performance levels within a discipline [21,33,34,37-39,48].

**Figure 2:**
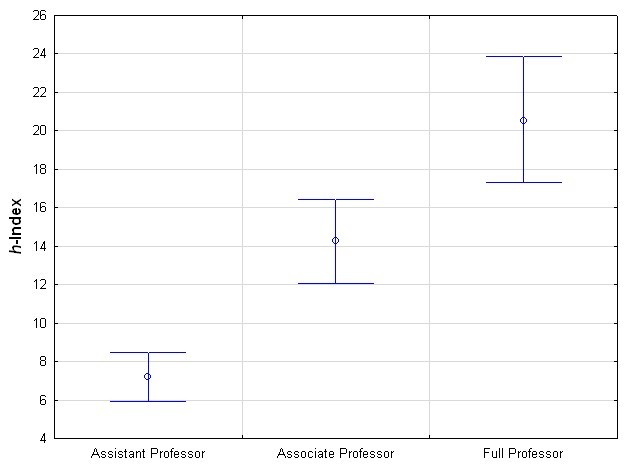
Mean *h*-index and 95% confidence interval for the three faculty ranks in US medical informatics departments.

We identified medical informatics departments in the United States using the list of medical informatics departments funded by the National Library of Medicine under the University-based Biomedical Informatics Research Training Programs [84], augmented with medical informatics departments listed in the Open Directory Project [85]. For each department we manually identified all faculty at any of the three ranks listed on their websites: assistant professor, associate professor, and full professor, for a total of 463 individuals. We then selected using simple random sampling 50 from each rank and computed their mean *h*-index and the confidence interval for the mean. The results are shown in Figure 2. There is greater variation in performance as the rank increases.

Given that the confidence intervals for the three ranks do not overlap, the differences in the mean *h*-index are statistically significant. Furthermore, our PR10 benchmark levels track the mean values by faculty rank well as seen in Table 5. This provides validation that the PR10 benchmark can be a useful tool for assessing the scientific performance of medical informatics faculty. For example, full professors in level 10 (top decile) on our PR10 benchmark would be above the average for US medical informatics faculty. On the other hand, a researcher with an *h*-index of 4 would be on level 5 of the PR10 benchmark and would therefore be below the mean for an assistant professor. However, such a level could be considered an acceptable target for someone completing a postdoctoral fellowship, for instance.

These results also indicate that the PR6 benchmark does not track medical informatics faculty rank very well, since both associate and full professors would be within the same level. This is due to PR6 making finer distinctions at the top end of the distribution and coarser distinctions otherwise. This results in multiple faculty ranks grouped into the same performance level. Therefore, one can argue that for practical purposes the PR10 benchmark is a more-useful tool for assessing and tracking performance of medical informatics academic faculty.

**Table 5:** Benchmark levels based on the mean *h*-index for the three US medical informatics academic faculty ranks.

Rank of faculty in US medical informatics departments	PR6 level^a^	PR10 level^b^
Assistant professor	2	6
Associate professor	3	8
Full professor	3	9

^a ^6 Percentile-level benchmark.

^b ^Decile benchmark.

### Interpretation and Use of the Benchmarks

Although medical informatics researchers come from multiple disciplines, the PR10 benchmark applies to the subset of individuals from these disparate disciplines who do medical informatics research. If the vast majority of medical informatics researchers were basic scientists, for example, then the benchmark would tilt more toward the performance of basic scientists. Similarly, if the vast majority of medical informatics researchers were computer scientists, then the benchmark would tilt toward the publication performance of that community.

The benchmark would be meaningful only for researchers whose area of research is clearly medical informatics. For example, a clinical researcher may have a high ranking on PR10, but this would not be relevant unless that individual has medical informatics as a primary area of work.

We suggest three scenarios where the PR10 benchmark can be useful. Researchers at early stages of their careers would be expected to be at the lower levels on the benchmark and to progress to higher levels over time. Therefore, the benchmark can be used as a yardstick to track performance over time. For research leaders, administrators, or funding agencies evaluating researchers at the same career stage competing for positions, funding, or promotions, the PR10 benchmark can be used to assess their relative standing in terms of scientific output. For instance, researchers with scores of 6 and 7 are in the same decile and may be considered equal on the *h*-index metric. Finally, the PR10 benchmark can be used to set objective gates (see Table 5), such as for hiring or promotion decisions.

In practice, the PR10 benchmark could replace or augment metrics such as the number of published papers or number of citations when assessing performance. It should not be used as the sole method for evaluating the publication performance of medical informatics researchers, but can serve as another useful input for such an evaluation. Furthermore, the PR10 benchmark would need to be updated on a regular basis to ensure that it reflects contemporary performance levels in the field.

### Limitations

An underlying assumption of our method is that medical informatics researchers will at some point publish in medical informatics journals (they only need to publish once in a top medical informatics journal to be in our sampling frame). For example, an author who has published medical informatics papers only in general medicine or general science journals, or who has published in conferences only but never in a top medical informatics journal, would not be in our sampling frame. Such an individual, however, would also likely not be considered to have medical informatics as a primary area of his or her research.

Our results are limited by the journals we selected from which to sample researchers. It is possible that a different set of journals would have produced different values for the benchmarks because they would have included a different group of researchers. However, we have argued that our choice of journals balances representativeness of the community and covers authors who publish influential work in the field.

While we used Scopus to compute our benchmark, one can argue that the use of another tool, such as Web of Science, may have produced different results. For example, Scopus indexes publications only since 1996. This would not account for citations to earlier research articles. On the other hand, medical informatics is a recent discipline, with JAMIA starting publication in 1994 and JMIR in 1999. Furthermore, there is evidence that Web of Science and Scopus produce very similar citation counts [86], which would also mean very similar *h*-index values.

## Multimedia Appendix 1

A critical review of the h-index and its measurement.

## Abbreviations

ACMI: American College of Medical Informatics
JAMIA: Journal of the American Medical Informatics Association
JMIR: Journal of Medical Internet Research
MeSH: Medical Subject Headings
PR6: 6 percentile-level benchmark
PR10: decile benchmark
