# Supplementary material for: Two h-Index Benchmarks for Evaluating the Publication Performance of Medical Informatics Researchers
Source: J Med Internet Res. 2012 Oct 18;14(5):e144. doi: 10.2196/jmir.2177 (PMC3517369; doi:10.2196/jmir.2177)
Supplement: Supplementary file 1 [file jmir_v14i5e144_app1.pdf]

Appendix A:
A Critical Review of the *h*-Index and its Measurement

1 Evaluations of the *h*-Index

Two recent systematic reviews have compiled much of the large amount of materials published on the *h*-index [1, 2]. Between 2005 and 2010 there had been close to 200 papers published on the subject [1]. These papers have evaluated the metric as well as proposed variations and alternatives meant to address its shortcomings. We have highlighted some of its benefits in the introduction. Below we summarize critiques of the *h*-index.

Criticisms of the *h*-index include that it is a single measure and consequently could result in an unfair rating of an individual. Therefore, other factors should be taken into account in the evaluation of an individual as there can be exceptions to the rule [3]. For example, the *h*-index is insensitive to the impact of an author with a small number of highly cited papers [1-3], which has been seen as both an advantage and a disadvantage of the metric.

The *h*-index has also been criticized because authors could use self-citation to inflate their *h* values. However, there have been many studies on this issue and overall, it has been shown that removing self-citations does not impact *h* values greatly while making the task of calculating *h* much more difficult. One of the largest impacts of self-citation was found by Kosmulski in his study of chemistry professors [1, 4], where he found that on average, self citations increased a professor's *h*-index scores by 26%. Self-citation also seems to have a greater effect on the *h*-index values of younger scientists [1]. On the other hand, it has been argued that within a field, self-citation habits are expected to be more or less uniform [1]. Therefore, no one author within a given field should have a great advantage over the others. Also, the impact of self-citation was shown to decrease over time as external citations for an author's work accumulate [1, 5].

The *h*-index has been said to be unfair to early career researchers, as their scores will invariably be low for a period of time as they build up their body of work. On the other hand, a strong correlation has been found between the original *h*-index and variants adjusted for age and career stage [6]. Similarly, the *h*-index has been shown to be biased against those who have interruptions in their work and those who work only part-time [1]. As a result, a gender bias has been found owing to the fact that women generally produced fewer papers than men due to such interruptions and/or reduced work schedules [1].

Another criticism of the *h*-index is that it is insensitive to the number and ranking of co-authors [1]. All co-authors for a given paper receive the same *h*-index value for that paper, from the primary author(s) to the last author in the list. Some have proposed methods to correct for this insensitivity. For example, Egghe proposes two methods for dealing with the co-author issue: (a) “fractional citation counts” and (b) “fractional paper counts” [7]. Methods such as these are useful in lessening the effect that papers with many co-authors would have on any individual author’s *h* score, but could undervalue the contributions of first authors who may have been more involved in the work than subsequent co-authors, and introduces the practical difficulty of quantifying individual contributions to a paper.

2 Citation Databases

The source of the citation counts used to calculate the *h*-index is integral to its accuracy [3]. Currently, the three most common interdisciplinary databases used to calculate the *h*-index are Web of Science (WoS), Scopus, and Google Scholar (GS) [1]. Results of the *h*-index vary between these databases because the sources they index also vary, and some benefits and drawbacks have been found for each [1].

Let us begin with GS, a relatively new resource for finding citations introduced in 2004. In terms of benefits, GS indexes a wide range of sources, some of which are not covered by Scopus and WoS, such as books, theses, reports, some conference proceedings, and pre-prints [8]. This can be important for some disciplines such as engineering, computer science and mathematics where such

publications are more common [1, 9-12]. GS could be used to supplement materials found in Scopus and/or WoS for authors who published in formats other than journal papers. However, GS indexes only materials that are digitally available and therefore misses many relevant references and citations, such as older works which have not yet been converted to digital format [10]. Furthermore, GS may not be able to access some publishers' electronic data, limiting its coverage [8, 13, 14]. The evidence is inconsistent as to whether GS finds more citations than WoS and Scopus [14-16], and the explanation for such inconsistencies is likely discipline specific [17]. While a strong correlation in citation counts was reported between WoS and GS [17], Meho and Yang found that the overlap between GS and the union of WoS and Scopus was 30.8%, and that GS was lacking 40.4% of the citations found by the other two databases [10]. Another study reported that GS lacked between 47% and 50% of the citations which were found by the other databases [16]. Many authors have also found that the citation counts in GS are inflated by duplicate citations, false positives and unscholarly citations [1, 9, 10, 12]. In order to ensure an accurate rating, citations should be verified to weed out any extraneous records [14]. This can sometimes be an arduous process, as Meho and Yang found out when they spent some 3,000 hours verifying GS citations in their study of LIS faculty [10].

Scopus is also a recent addition, launched by Elsevier in 2004. Scopus has been held up as an excellent resource for calculating the *h*-index [1, 2, 18]. According to its *Content Coverage Guide,* Scopus currently holds over 44 million records, indexed from 18,500 active serial titles, including peer-reviewed journals, trade journals, book series, and conference materials [19]. Jacso, in his study of the calculation of the h-index across the three databases, indicates that Scopus has “the best software module for presenting results lists” as it sorts articles by decreasing citation counts [18]. Jacso also asserts that Scopus has the most accurate automated calculation of the *h*-index [18]. The automated calculation in Scopus may still exclude some citations that cannot be properly matched by the software, but it has been found to be more accurate than the WoS automated *h*-index calculator [18]. However, Scopus is limited in that it only indexes cited

references from 1996. It also does not count citations for materials published prior to 1996 that have been enumerated since that time [1]. Therefore, for scholars who published prior to 1996, results in Scopus may not be complete. Meho and Yang found that for the group they were studying, the overlap between citations found in WoS and Scopus was not considerable, 58.2%, and that Scopus had a higher percentage of unique citations than did WoS (26% vs. 15.8%) [10]. Another study found that the overlap in citations between Scopus and WoS was between 41% and 59%, depending on the discipline [16]. Scopus was found to have greater coverage of conference proceedings than WoS, which may account for much of the difference between these two databases [10]. However, we should note that WoS has expanded its coverage of conference proceedings since Meho and Yang's study was conducted.

WoS originated from the *Science Citation Index* developed by Eugene Garfield in the early 1960s [20]. Now part of the Thomson ISI Web of Knowledge product, it has been a trusted resource for academics over the years [3]. Jacso holds WoS above all other databases as the most complete [18]. Currently, the Thomson Reuters website indicates that WoS content covers 12,000 journals and 150,000 conference proceedings dating back as far as 1900, for a total of over 46 million records [21]. It has been found, however, that in order to obtain accurate citation counts in WoS, one must seek out and identify all stray and orphan citations that could not be properly matched by its software [1, 9, 18]. The automatic *h*-index calculations, as well as citation searches by author, can exclude many citations that are integral to the *h*-index score of an individual [1]. Jacso found that the *h*-index calculated in WoS doubled from the automated version to the manually verified version [18]. WoS indexes ISI journals and therefore seems to have a wider range of sources relating to the natural sciences than other disciplines such as computer science [9]. Consequently the results will vary depending on the discipline [9]. Although WoS is held up as a gold standard of sorts, and contains the widest range of historical records, it is susceptible to similar problems and limitations as Scopus and GS. Jacso warns, "Corroborative tests

must be done in every database for important research whose results may affect people" [12].

It could be argued that a union of results from all databases may present the most complete picture of an individual's impact, but does combining citation counts from different databases affect *h*-index scores in a significant way? Combining the citations for faculty in library and information science departments from WoS and Scopus significantly altered the ranking of individuals who "appear in the middle of the rankings", but when looking at the group as a whole the overall ranking did not change significantly [10]. In addition, adding GS citations increased the total number of citations for the group by 93.4%; however, these citations did not significantly change the ranking of the group members [10]. One study found very high correlations between GS and Scopus *h*-index values for US neurosurgeons [22]. Another study found that *h*-index results were ≥30% higher when calculated using GS for individuals in the areas of mathematics and computer science [11]. Therefore, the impact of citations from GS on *h*-index values may be discipline specific. Unfortunately, we are not aware of attempts to combine citations from the three databases so the effects of such a compilation are not known in this case. However, to the extent that rankings are not affected, the computation of percentiles in our benchmarks should not be affected by the choice of database.

3 References

1. Norris M, Oppenheim C. The h-index: A broad review of a new bibliometric indicator. Journal of Documentation, 2010; 66(5):681-705.
2. Alonso S, Cabrerizo FJ, Herrera-Viedma E, Herrera F. h-Index: A review focused in its variants, computation and standardization for different scientific fields. Journal of Informetrics, 2009; 3(4):273-289.
3. Hirsch JE. An index to quantify an individual's scientific research output. Proceedings of the National Academy of Sciences of the United States of America, 2005; 102(46):16569-16572.[10.1073/pnas.0507655102]
4. Kosmulski M. A new Hirsch-type index saves time and works equally well as the original h-index. ISSI Newsletter, 2006; 2(3):4-6.
5. Engqvist L, Frommen J. The h-index and self-citations. Trends Ecol Evol., 2008; 23(5):250-2. Epub 2008 Mar 25.
6. Lee J, Kraus KL, Couldwell WT. Use of the h index in neurosurgery. Clinical article. J Neurosurg, 2009; 111(2):387-92.[10.3171/2008.10.JNS08978]

7. Egghe L. Mathematical theory of the h- and g-index in case of fractional counting of authorship. J. Am. Soc. Inf. Sci. Technol., 2008; 59(10):1608-1616.[10.1002/asi.v59:10]
8. Kousha K, Thelwall M. Sources of Google Scholar citations outside the Science Citation Index: A comparison between four science disciplines. Scientometrics, 2008; 74(2):273-294.[DOI 10.1007/s11192-008-0217-x]
9. Harzing AWK, van der Wal R. Google Scholar as a new source for citation analysis. Ethics in Science and Environmental Politics, 2008; 8(1):61-73.[10.3354/esep00076]
10. Meho LI, Yang K. Impact of data sources on citation counts and rankings of LIS faculty: Web of Science versus Scopus and Google Scholar. J. Am. Soc. Inf. Sci. Technol., 2007; 58(13):2105-2125.[10.1002/asi.v58:13]
11. Bar-Ilan J. Which h-index? — A comparison of WoS, Scopus and Google Scholar. Scientometrics, 2008; 74(2):257-271.
12. Jacso P. The pros and cons of computing the h-index using Google Scholar. Online Information Review, 2008; 32(3):437 - 452.
13. Jacso P. Google Scholar: the pros and the cons. Online Information Review, 2005; 29(2):208-214.[Doi 10.1108/14684520510598066]
14. Bornmann L, Marx W, Schier H, Rahm E, Thor A, Daniel HD. Convergent validity of bibliometric Google Scholar data in the field of chemistry-Citation counts for papers that were accepted by Angewandte Chemie International Edition or rejected but published elsewhere, using Google Scholar, Science Citation Index, Scopus, and Chemical Abstracts. Journal of Informetrics, 2009; 3(1):27-35.[DOI 10.1016/j.joi.2008.11.001]
15. Bauer K, Bakkalbasi N. An examination of citation counts in a new scholarly communication environment. D-Lib Magazine, 2005; 11(9).
16. Bakkalbasi N, Bauer K, Glover J, Wang L. Three options for citation tracking: Google Scholar, Scopus and Web of Science. Biomed Digit Libr, 2006; 3:7.
17. Kousha K, Thelwall M. Google Scholar citations and Google Web/URL citations: A multi-discipline exploratory analysis. Journal of the American Society for Information Science and Technology, 2007; 58(7):1055-1065.[Doi 10.1002/Asi.20584]
18. Jacso P. Testing the Calculation of a Realistic h-index in Google Scholar, Scopus, and Web of Science for F. W. Lancaster. Library Trends, 2008; 56(4):784-815
19. SciVerse Scopus. SciVerse Scopus Content Coverage Guide. 2011, Elsevier B.V.
20. Fingerman S. Web of Science and Scopus: Current Features and Capabilities. Issues in Science and Technology Librarianship. 2006; Available from: [http://www.istl.org/06-fall/electronic2.html]. Accessed on: January 1, 2012.
21. Thomson Reuters. Products A-Z: Web of Science. Available from: [http://thomsonreuters.com/products_services/science/science_products/a-z/web_of_science/#tab2]. Accessed on: October 11.
22. Lee J, Kraus K, Couldwell W. Use of the h index in neurosurgery. Journal of Neurosurgery, 2009; 111:387-392.
